# Recent advances in the characterization of essential genes and development of a database of essential genes

**DOI:** 10.1002/imt2.157

**Published:** 2024-01-02

**Authors:** Ya‐Ting Liang, Hao Luo, Yan Lin, Feng Gao

**Affiliations:** ^1^ Department of Physics Tianjin University Tianjin China; ^2^ Frontiers Science Center for Synthetic Biology and Key Laboratory of Systems Bioengineering (Ministry of Education) Tianjin University Tianjin China; ^3^ SynBio Research Platform Collaborative Innovation Center of Chemical Science and Engineering (Tianjin) Tianjin China

**Keywords:** database of essential genes, drug and vaccine design, essential gene, gene essentiality prediction, genome design

## Abstract

Over the past few decades, there has been a significant interest in the study of essential genes, which are crucial for the survival of an organism under specific environmental conditions and thus have practical applications in the fields of synthetic biology and medicine. An increasing amount of experimental data on essential genes has been obtained with the continuous development of technological methods. Meanwhile, various computational prediction methods, related databases and web servers have emerged accordingly. To facilitate the study of essential genes, we have established a database of essential genes (DEG), which has become popular with continuous updates to facilitate essential gene feature analysis and prediction, drug and vaccine development, as well as artificial genome design and construction. In this article, we summarized the studies of essential genes, overviewed the relevant databases, and discussed their practical applications. Furthermore, we provided an overview of the main applications of DEG and conducted comprehensive analyses based on its latest version. However, it should be noted that the essential gene is a dynamic concept instead of a binary one, which presents both opportunities and challenges for their future development.

## Highlights

- Essential genes are crucial for organism survival and development, which have been identified using various experimental methods across different organisms, providing valuable resources for studying their characteristics and developing predictive algorithms.
- The database of essential genes (DEG) collects experimental results on essential genes and serves as a widely used resource for studying them. It enables quick access to essential gene information for specific species and has applications in essential gene analysis, prediction, drug and vaccine design, and artificial genome construction.
- The definition of essential genes is context‐dependent, not a simple binary classification. Integrating different experimental results of essential genes is challenging but offers opportunities to explore gene interaction mechanisms.

## Graphical abstract

Essential genes are indispensable for the survival and development of organisms, and have been successfully identified using various experimental methods across diverse organisms. DEG, a database of essential genes is dedicated to collecting experimental results on essential genes and has become one of the most commonly used tools for studying essential genes, where the information on essential genes for a specific species can be quickly accessed, with a broad range of applications, such as essential gene feature analysis and prediction, drug and vaccine design, as well as artificial genome design and construction. The definition of essential genes is environment‐specific rather than simple binary, which should not be overlooked in the study of essential genes.

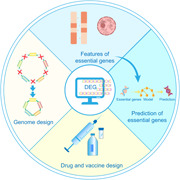

## INTRODUCTION

Essential genes are necessary for the survival of an organism under specific environmental conditions and play crucial roles in fundamental biological and genetic processes [1]. The gene essentiality is important in both theoretical and applied research [2, 3]. Therefore, it is vital to identify essential genes as this information can be applied in the fields of life sciences, pharmacology, and synthetic biology. However, gene essentiality is conditionally dependent on various factors, such as growth conditions, developmental stages, and genetic environment [4]. Different experimental conditions for the same organism may yield different results, which can change over the course of evolution [5]. Gene essentiality is a quantitative trait that must be evaluated continuously. Therefore, the effects of the essential genes under specific conditions should not be overlooked.

In general, the prediction of essential genes relies primarily on laboratory approaches. In 1999, the first set of essential genes in living organisms was determined using global transposon mutagenesis in *Mycoplasma genitalium* [6]. Subsequently, extensive research has been conducted on essential genes in a wide range of organisms. The development of experimental techniques for identifying essential genes has facilitated the collection of essential gene data. The most commonly used experimental methods for predicting essential genes include single‐gene knockout [7], RNA interference [8], antisense RNA [9], transposon mutagenesis [6], and clustered regularly interspaced short palindromic repeat‐associated protein (CRISPR)‐Cas9 [10, 11]. In addition to experimental methods, computational prediction methods have emerged to facilitate the identification of essential genes. The most commonly used computational methods include comparative genomics‐based, constraint‐based, and machine‐learning‐based prediction methods, which provide important references for future research on essential genes.

In recent years, studies on essential genes have been conducted in various fields. For example, understanding the functions of essential genes is essential for discovering the minimal core components of cells, which are crucial in synthetic biology [12]. Research on essential genes can accelerate the development of microorganisms with desired phenotypic traits [13], and facilitate the development of drugs and vaccines. Essential bacterial proteins can serve as potential drug targets for new antibiotics because their indispensable role in bacterial life renders them vulnerable to disruption [14]. Certain essential proteins conserved in specific species are considered promising candidates for broad‐spectrum drug targets, whereas others specific to a particular bacterium are viable candidates for species‐specific targets. Moreover, the essentiality of novel genes can be predicted by constructing models based on existing essential gene data.

With the increasing amount of essential gene data, databases, and online services based on experimental or predicted essential gene data have emerged, providing convenient and reliable references for essential gene‐related research. The database of essential genes (DEG) was established in 2004 and has been continuously updated with the accumulation of experimental data [15, 16, 17, 18]. This database compiles essential gene data obtained from a diverse range of experimental methods on a whole‐genome scale. To date, DEG has become an important reference for essential gene‐related research, with the database cited more than 1100 times based on Web of Science data.

In this review, we provide a brief overview of the technological advances in essential gene prediction, relevant databases, online services, as well as the applications of essential genes, and emphasize that the concept of gene essentiality is environmentally specific. In addition, we discuss the significant applications of DEG in providing researchers with reliable references. Furthermore, we propose potential directions for expanding the use of DEG.

### The definition of essential genes

Essential genes are indispensable for the survival of an organism and are, therefore, considered the foundation of life. Nonessential genes refer to the genes that have been experimentally determined to be dispensable for the growth of an organism, rather than the genes other than the essential ones. However, the estimated proportions of essential genes vary significantly among species and studies [19]. Diverse studies within the same species have shown that genes may be essential in one strain but not in another [5, 20] or that genes may be essential for one growth condition but not for others [21, 22], suggesting that essentiality is not an intrinsic property of genes. The reasons for such variations are diverse, including differences in experimental methods, conditions, and even the influence of experimental errors. Hence, the term “essential gene” is highly dependent on context. Only when the environment for the survival of an organism is precisely defined can a gene be classified as essential. The dependence of a cell on specific genes/gene products is influenced by its external environment and genetic context, including the presence or absence of other genes/gene products [23]. As a result, the essentiality of a gene can vary depending on these factors and change with each deletion. These genes are referred to as “conditionally essential genes” [24]. For example, some genes have been identified as “protective essential” as they can become dispensable with the removal of another gene, subsequently rendering them nonessential. This is typically because the former encodes a protective function against the toxic effects of the latter gene [25, 26, 27]. Conversely, the loss of a second gene may render a nonessential gene essential (synthetic lethality) [28]. Synthetic lethality was originally discovered in studies involving fruit fly and yeast, where individual inactivation of either of the two genes had minimal impact on cell viability. However, simultaneous disruptions in the expression of two genes or multiple independent genes, including mutations, overexpression, and gene suppression, can lead to cell death [29, 30]. Furthermore, gene products can form complexes in which nonessential genes contribute to essential functions. For instance, among the protein‐coding genes in budding yeast involved in reproduction, five groups of nonessential genes that potentially encode proteins with fundamental functions have been identified [31]. Nutritional conditions can also influence essential genes, as mutant strains carrying inactivated nonessential genes may exhibit minimal or negligible effects on cell phenotypes under optimal conditions and severe impairment or loss of viability under suboptimal conditions [32, 33]. However, evidence suggests that many nonessential genes grown in nutrient‐rich media are important for adaptability to alternative growth conditions [34]. Additionally, different experimental methods may yield different outcomes. For example, CRISPR‐based approaches have identified more essential genes in human cell lines compared to RNA interference (RNAi)‐based methods [35, 36]. Considering these factors, essentiality may be a quantitative characteristic rather than a simple essential/nonessential classification, necessitating standardized quantitative approaches [37]. Research indicates that dispensable essential genes often exhibit characteristics akin to those of nonessential genes. This is attributed to the abundance of genes with paralogous counterparts compared to other essential genes, lower levels of co‐expression, and the near absence of genes encoding protein complex components. This outcome explains the distinction between essential dispensable and permanently essential genes. Moreover, based on these features, a random forest prediction model has been developed for the identification of conditionally essential genes [38].

Recent studies have found that bacteria and yeast can adapt through genomic changes and adaptive mutations that restore cellular function in response to the inactivation of conditionally essential genes [39, 40]. These studies suggest that, despite the loss of essential gene function, the essentiality of genes depends on the cell's capacity to acquire mutations and restore proliferation. This indicates that essentiality is no longer solely a gene attribute, but rather a cellular attribute, attributing essentiality to cellular pathways rather than the genes themselves [41]. These findings present new research directions and challenges for defining and studying essential genes.

### The identification of essential genes

**Figure 1:**
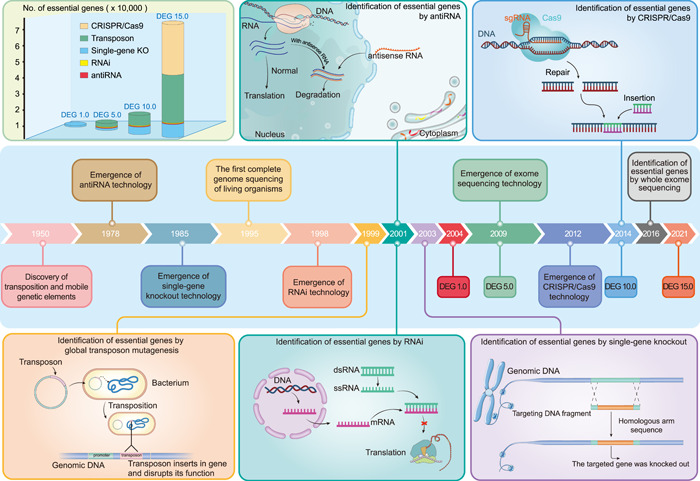
Milestones of technological breakthroughs in gene essentiality. It illustrates some of the important technological advancements and their initial applications in essential gene research. The update of different versions of the database of essential genes (DEG) and the data volume obtained through different methods in each version of DEG is also indicated.

The identification of essential genes is a long‐standing question in the field of molecular biology. Currently, there are two main approaches for determining essential genes: experimental and computational. Experimental methods can provide specific results for essential genes under different experimental conditions (Figure 1). However, they can be expensive and labor‐intensive. As a result, various computational methods have been proposed to predict essential genes using computers with the aim of alleviating these limitations.

### Experimental‐based methods

Identification of essential genomes has a long history in the field of molecular biology. In 1951, Horowitz and Leupold proposed that some major components of proteins may be essential for life [42]. Before the “genomics era” early mutagenesis techniques involved the induction of random mutations in the genome of organisms using chemical or physical agents [43]. Essential genes were predicted by inducing random chemical mutagenesis and analyzing progeny survival. For example, studies have shown that approximately 50%, 15%, and 12% of the genomes of *Drosophila melanogaster*, *Caenorhabditis elegans*, and *Saccharomyces cerevisiae* are essential [44, 45, 46, 47]. Nonetheless, the experimental techniques at that time were insufficient to identify specific essential genes. In the late 20th century, transposon techniques [48, 49], single‐gene knockout [50], antisense RNA [51], and RNA interfaces [52] were developed, providing more possibilities for research focused on the gene level. In 1995, the first complete genome sequences of *Haemophilus influenzae* and *M. genitalium* were obtained by using high‐throughput [53, 54]. The complete genome sequence of an organism is a prerequisite for acquiring a complete list of genes. The complete genomes of model organisms such as *Escherichia coli*, *Bacillus subtilis*, and *S. cerevisiae* were published in the subsequent 2 years [55, 56]. The first experiment to identify the essential gene set of an organism was conducted in 1999, which confirmed the minimal gene set required for the survival of *M. genitalium* using whole‐genome transposon mutagenesis [6]. The principle of transposon insertion for detecting essential genes is based on the random insertion of transposons and their effects on gene disruption. By analyzing the insertion site and expression of transposon‐disrupted genes, essential genes and their effects on growth can be determined. Subsequently, methods have been developed to identify essential genes by targeting single‐gene knockout [7], RNAi [8], and antisense RNA [9], thereby enabling researchers to explore the essentiality of genes in various organisms. Single‐gene knockout experiments involve the removal of one or more genes to observe phenotypic changes. For genome‐wide studies, this process must be repeated several times, which necessitates comprehensive genome annotation. RNAi technology identifies essential genes by introducing double‐stranded RNA molecules that are complementary to the messenger RNA (mRNA) of a target gene, leading to mRNA degradation and inhibition of protein expression. Antisense RNA technology identifies essential genes by introducing single‐stranded RNA molecules that are complementary to the mRNA of the target gene, leading to the inhibition of gene expression.

In the early stages of essential gene research, the focus was primarily on microorganisms. Efforts to target gene disruption in animals were hindered by the inherent inefficiency of homologous recombination in animal tissue cultures [57]. The first breakthrough was the discovery that homologous recombination is more effective in embryonic stem cells derived from mouse blastocysts, leading to the development of the first knockout mouse in 1989 [58]. In 2003, the first genome‐wide RNAi screen was conducted on the nematode *C. elegans* to systematically define mutant phenotypes [59]. Subsequently, this technology has been rapidly applied to mammalian cells, and several groups have generated RNAi libraries encompassing human and mouse genomes for gene importance screening [60, 61]. RNAi has become the primary method for studying essential genes in mammals. However, off‐target effects and incomplete gene function limitations cannot be ignored.

The advent of next‐generation sequencing technologies has enabled the rapid acquisition of whole‐genome sequences of various species. Convenient access to sequencing data has facilitated the introduction of other methodologies for handling genetic variations. Transposon sequencing (Tn‐seq) is an emerging technology that combines transposon mutagenesis with high‐throughput sequencing and utilizes a high‐density transposon insertion library constructed in the target organism to perform functional analysis of its whole genome through high‐throughput sequencing [62]. Tn‐seq techniques such as TraDIS [63], INSeq [64], HITS [65], and the Tn‐seq Circle [66] have been extensively used for essential gene detection in microorganisms. The application of Tn‐seq has enhanced the understanding of essential genes by incorporating essential genomic elements, including noncoding RNAs, rather than focusing solely on protein‐coding genes. In addition, Tn‐seq can be used to screen essential genes under various experimental conditions, both in vitro and in vivo, and is not just limited to culture conditions [67]. Tn‐seq significantly increases the number of essential genes identified under different conditions.

Whole‐genome essentiality screens have elucidated the molecular basis of several biological processes. However, RNAi‐based screens are often confounded by off‐target effects and gene knockdown rather than complete loss of function, limiting our knowledge of essential genes in human cells [68]. However, the emergence of the CRISPR/Cas9 system has revolutionized mammalian cell genome editing [69, 70]. At its core, the programmable DNA endonuclease is composed of two components: the Cas9 protein derived from *Streptococcus pyogenes* (or similar proteins from other species), and a single guide RNA that guides endonuclease activity to the target DNA sequence [71]. CRISPR induces sequence‐specific DNA double‐strand breaks, leading to frameshift insertions/deletions that result in the complete loss of protein function [72]. This technology has made cost‐effective and efficient gene editing possible in yeast, plants, and animals with a significant impact on human cell editing [73, 74, 75]. Additionally, the catalytically dead version of Cas9 (dCas9) can be employed to target specific DNA sequences with single guide RNA, which is known as CRISPR interference (CRISPRi), or to activate gene expression, which is referred to as CRISPR activation (CRISPRa) [76]. Cas9 and dCas9 have been used to map the composition of important components in human cell lines [77]. In 2015, three papers simultaneously reported the genome‐wide identification of essential genes in different human cell types [78, 79, 80]. In addition, whole exome sequencing is another breakthrough that captures and enriches the DNA of exonic regions of the entire genome using either sequence capture or targeted amplification techniques [80]. Compared to whole‐genome resequencing, whole‐exome sequencing mainly targets gene sequences in exonic regions with deeper coverage and higher data accuracy to quickly identify essential human genes in vivo [81].

### Computational‐based methods

Considering the complexity, high cost, labor, and time intensiveness of experimental methods, computational approaches have been deployed as a supplement to experimental techniques to minimize the resources required for essentiality analysis. As the number of essential genes identified through experiments increases, reliable references are being provided to develop methods for essential gene prediction. In general, three approaches are employed to determine gene essentiality: comparative genomics‐based, constraint‐based, and machine‐learning‐based methods.

Initially, essential gene prediction was conducted using comparative genomics‐based methods that rely on homology [82]. Homology mapping occurs between genes that are duplicated within an organism (paralogs) or between related genes in two or more different organisms (orthologs) that are products of speciation. Homology mapping involves comparison of the sequences of two organisms and determination of their sequence similarity based on a defined percentage identity threshold. This method has been used to predict core genes in bacterial species such as *Mycoplasma* spp., *Corynebacterium* spp., *Plasmodium falciparum*, and *Brucella* spp. [83, 84, 85, 86].One major challenge is the significant impact of evolutionary distance on the results of comparative genomic analysis. Although essential genes are often highly evolutionarily conserved, especially in bacteria, conserved genes across species are not always essential [87], which render homology‐based methods less effective in predicting essentiality. Previous large‐scale analyses have shown that only a small number of genes are conserved across the tree of life, implying that many essential genes are species‐specific [88].

Constraint‐based prediction methods utilize genome‐scale metabolic networks to elucidate the biology of the metabolic pathways within an organism. This approach relies on constraint‐based modeling techniques applied to reconstructed metabolic networks based on genome sequencing and annotation to study the structure, function, and interactions of the network [89]. Flux balance analysis (FBA) is the most widely used constraint‐based method for analyzing metabolic network properties. It predicts the fluxes of metabolites under steady‐state conditions by applying mass balance constraints to stoichiometric models [90]. Using FBA to predict essential genes involves simulating gene knockouts and assessing their impact on a network [91]. Constraint‐based models have been constructed in organisms from all three domains of life and have facilitated the study of gene essentiality [92]. The FBA has a low computational cost because it does not require kinetic parameters. However, FBA has notable limitations. First, it can only predict the importance of metabolic genes [93]. In addition, unlike the ability to combine steady‐state and dynamic analyses, FBA requires enzyme kinetic data to evaluate the activity of genome‐scale metabolic reactions under transient conditions [94]. Finally, FBA often requires enzymatic reactions to address the limitations in metabolic models, which may sometimes be inconsistent with experimental data. This depends on empirical modeling, and in some cases, parameter prediction can be challenging [95].

Currently, the most widely used essential gene prediction method is based on the construction of machine learning algorithms, using features derived from the analysis of experimental results on essential genes. Generally, the features of essential genes can be divided into two categories: sequence‐ and context‐related (Table 1).

**Table 1:** Summary of characteristics of essential genes.

Category	Feature	Description	References
Sequence features	Expression level	Studies have shown that essential genes are expressed at higher levels than nonessential genes.	[96, 97]
	Codon bias	Essential genes are more likely to use optimal codons. Some characteristics of codons can serve as parameters for assessing the ideal codon usage, which plays a critical role in ensuring precise translation of highly expressed genes.	[98, 99]
	Protein size	Larger proteins have more biological functions and are more conserved, it is believed that larger proteins tend to be enriched in essential proteins.	[100]
	Gene position	There is a higher proportion of essential genes located in operons and the leading strand.	[101]
	Subcellular location	Essential proteins are found predominantly in the cytoplasm, although significantly greater proportions of nonessential proteins are situated in other cellular areas.	[100, 102]
	Hurst exponent	Essential genes have a lower average Hurst exponent.	[103]
Context‐related features	Evolutionary relationships	Essential genes evolve more slowly than nonessential genes.	[104, 105]
	Protein function	Essential genes are hubs of protein–protein interaction networks, which are more commonly involved in fundamental categories according to gene ontology and Kyoto Encyclopedia of Genes and Genomes pathway.	[106, 107]
	Protein‐protein interactions	Essential genes tend to have higher connectivity in protein‐protein interaction networks.	[108, 109]
	Domain properties	Protein essentiality is to be conserved through the function of protein domains or domain combinations.	[110, 111]

Research on the characteristics of essential genes provides a reference for predicting essential genes using machine learning [112]. Typically, the development of predictive models in machine learning involves the following steps: feature selection (different features of essential genes), construction of training and testing datasets (essential/nonessential gene data), selection and design of machine learning algorithms, and evaluation of model predictive performance [113]. Various studies have used genomic and protein features to develop and train classifiers for predicting gene essentiality. Over the years, several algorithmic models based on genomic features have been successfully developed for the computational identification of essential genes. For example, Fan et al. developed an SCP algorithm that combines essential gene protein–protein interaction (PPI) and subcellular localization. This method integrates an improved PageRank algorithm based on gene expression data with weighted subcellular localization and the Pearson correlation coefficient of weighted subcellular localization [114]. Additionally, predictive models have emerged for essential genes in the noncoding regions. Zhang et al. developed an iEssLnc model using metapath‐guided random walks, which was the first estimation model for the essentiality of lncRNA genes [115]. It can be inferred that “good” data and efficient machine learning techniques are required for accurate prediction. Supervised, semi‐supervised, unsupervised, and reinforcement learning are commonly used machine learning techniques [116, 117]. However, gene essentiality prediction is often modeled as a classification problem under supervised learning.

Deep learning is a subset of machine learning in artificial intelligence, in which networks can learn from unstructured or unlabeled data in an unsupervised manner. Recently, deep learning has been used to predict essential genes. For example, Deeply Essential is a deep neural network that uses only sequence information to predict essential genes [118]. Compared with previous approaches using clustering and undersampled data sets, this model achieves higher sensitivity and accuracy [119]. Another deep learning model for essentiality prediction employs a different approach using a framework that automatically learns biological features without prior information. This network utilizes information on gene expression, subcellular localization, and PPI networks to learn topological features [120]. However, the two main drawbacks of applying deep learning to gene essentiality prediction are as follows: (i) deep neural networks require large amounts of data for training to outperform traditional machine learning algorithms and (ii) tuning hyperparameters in deep learning models is complex.

While the application of machine learning methods is convenient, it also faces challenges such as the difficulty of measuring the quality of predictions and the inability to generalize across specific experimental contexts. Furthermore, considering that the definition of essential genes is context‐specific, caution should be exercised in defining the criteria for training ML models for essentiality prediction, depending on the purpose of the study. Moreover, the selection of features and combinations may affect the predictive performance, and there is no definitive method for selecting appropriate features for different organisms [121]. Finally, for understudied species, the choice to study within‐species data is limited by the small number of known essential genes, whereas the use of cross‐species data may result in decreased accuracy.

### Related databases and web servers

Online databases of essential genes have been created for various species based on experimental data and computational models (Table 2). Researchers can use data from these databases to study the intrinsic characteristics of essential genes/proteins and uncover features closely associated with essentiality. In addition to the experimentally derived databases of essential genes, databases storing predicted essential gene data have also been established, along with some open‐access programs for conducting essential gene predictions.

**Table 2:** Description of essential gene databases and web servers.

Resource	Description	URL	References
OGEE v3	Database that provides essential and nonessential genes with factors known to contribute to gene essentiality	https://v3.ogee.info	[122]
EGGs	Database that provides essential genes for visualization and analysis on a subsystem diagram	http://www.nmpdr.org/FIG/eggs.cgi	[123]
DEG 15.0	Database of essential genomic regions based on experimental data with embedded BLAST tools	http://www.essentialgene.org	[18]
pDEG	Database of predicted essential genes in *Mycoplasma* species, which integrates biased distribution information of essential genes	http://tubic.org/pdeg	[124]
CEG 2.0	Database based on clusters of directly homologous essential genes, which was originally derived from DEG and OGEE	http://cefg.uestc.cn/ceg	[125]
NetGenes	Database that contains predicted essential genes for more than 2700 bacteria using features derived from PPI networks	https://rbc-dsai-iitm.github.io/NetGenes	[126]
ePATH	Database of predicted essential genes for more than 4000 organisms based on experimental data and functional KEGG orthologs	https://www.pubapps.vcu.edu/epath	[127]
CEG_Match	Web server developed based on the CEG database, which is a gene essentiality prediction tool based on its function	http://cefg.uestc.cn/ceg	[125]
Geptop 2.0	Web server that identifies essential genes in prokaryotes using ortholog definitions, incorporating best hits and weighting phylogenetic distances from DEG or OGEE	http://cefg.uestc.cn/geptop	[128]
ZCURVE 3.0	Web server used to predict genes in bacterial or archaeal genomes, which is developed based on the Z‐curve theory	http://guolab.whu.edu.cn/ZCURVE/	[129]
EGP	Web server that predicts essential genes in bacteria using SVM, incorporating 16 independent feature sets for model construction	http://cefg.uestc.edu.cn:9999/egp	[130]

Abbreviations: DEG, database of essential genes; KEGG, Kyoto Encyclopedia of Genes and Genomes; PPI, protein–protein interactions.

### The practical applications of essential genes

#### Applications in the field of synthetic biology

Essential genes are responsible for maintaining normal physiological and metabolic processes in cells, making them essential for constructing cells with high stability or specific functions [23]. Therefore, these genes provide a theoretical basis for relevant research such as genome design. Minimal genomes are currently one of the best proofs of concept in synthetic biology. A minimal genome refers to the genome required to maintain the most basic life activities of an organism; it has been a crucial research aim in the field of synthetic biology [15]. Establishing a minimal, universal set of genes required to sustain life can considerably enhance our understanding of life at the most basic level and also has practical applications in production. As cornerstones of synthetic biology, the essential genes could serve as references for constructing a minimal genome. Notably, the concepts of essential genes and minimal genomes are not completely equivalent. The essential genes represent the set of genes required for successful reproduction, whereas the minimal genome represents genes necessary to maintain cell viability [131]. In practical, the identification and investigation of essential genes are typically employed to infer the composition of the minimal genome. However, computational modeling of bacterial metabolic networks has demonstrated that a minimal genome requires a greater number of genes than all essential genes combined [132]. For constructing a minimal genome, the top‐down approach and bottom‐up approach have been proposed [133, 134, 135] The top‐down approach reduces the genome size by deleting randomly selected or unidentified genomic segments. Deletions can be accomplished through various experimental approaches, such as plasmid‐ and linear DNA‐based methods, as well as the utilization of site‐specific recombinases, transposons, and the CRISPR/Cas system [133, 136]. Through a top‐down approach of progressively removing nonessential genes and functional elements, several examples of genome minimization have been achieved [3, 137, 138]. Additionally, a top‐down genome deletion algorithm called MinGenome was proposed, which starts with the longest possible deletions and consecutively eliminates metabolic and regulatory genes. To avoid lethal or growth‐defective deletions, this algorithm retains essential genes and synthetic‐lethal pairs by imposing constraints on biomass production [139]. The main advantage of the top‐down approach is that it begins with an operable genome, allowing any detrimental effects caused by deletions to be remedied by reverting to the latest nondeleted state. However, this method is time‐consuming and may lead to unforeseen dead ends, because the genetic landscape is altered at each step of the process, influencing the importance of other genes.

The bottom‐up approach connects gene fragments to specific functions. Advancements in DNA synthesis, sequencing technologies, and genome transplantation have enabled the synthesis of long DNA sequences with complex gene compositions. The primary method utilized for this is polymerase chain reaction technology, which enables the assembly of overlapping pools of short oligonucleotides and provides a technical foundation for bottom‐up approaches [140]. Based on several rounds of rational design and random mutagenesis of gradually reduced genomes, an approximate minimal bacterial genome, JCVI‐syn3.0, which contained 98 additional genes compared to the initially predicted set of essential genes in individuals, was constructed. This observation can be attributed to the fact that nonessential genes in the original genome become essential or quasi‐essential because of synthetic lethality during genome reduction [141]. Breuer et al. utilized the accumulated metabolic data from mycoplasmas and other bacteria, and applied it to a flux balance analysis model to establish a computational model of the JCVI‐syn3.0 metabolic network, which could better predict essential and quasi‐essential genes in JCVI‐syn3.0 [142]. During the bottom‐up design process, it is necessary to elucidate the complete genetic information for each gene and its interactions within the entire genetic background. Owing to a limited understanding of the principles of genome design and the complexity of the target organism, even for bacteria with smaller genomes, numerous possible genome configurations pose challenges for this approach [143].

As mentioned above, one of the major difficulties in constructing a minimal genome is elucidating the interactions between genes within the entire genetic background, which poses significant challenges for the identification of essential genes and genome construction in the early stages. However, computer‐assisted methods can accelerate the generation of large‐scale data corresponding to genetic content and cellular functionality by characterizing cells with various types of genome modifications, and thus has the potential to broaden and deepen our understanding of the entire cellular system and contribute to the production of industrially valuable biological systems.

#### Applications in the field of medicine

The traditional drug and vaccine discovery methods are resource‐intensive and time‐consuming. Subtractive genomics and reverse vaccinology have recently been classified as powerful approaches for identifying drug and vaccine candidates [144, 145, 146]. These approaches have streamlined drug development by eliminating the need for costly and time‐consuming trial‐and‐error experiments. The identification of potential targets is the first step in drug and vaccine discovery. Considering that the absence or inhibition of essential genes can have lethal effects on microorganisms, essential genes can be used as screening criteria for drug and vaccine targets in subtractive genomics and reverse vaccinology [14]. In addition, in the field of cancer treatment, the concept of synthetic lethality has been extended to paired genes, where one gene is inactivated by a deletion or mutation, and pharmacological inhibition of another gene leads to cancer cell death, while normal cells are not affected. In the most direct application, a targeted therapy could be determined to kill cancer cells lacking specific tumor suppressor genes but retain normal cells [24]. Moreover, the genes essential in cancer cell lines but nonessential in human tissues can reveal oncogenic drivers, paralog expression patterns, and chromosomal structures associated with the corresponding cancer types [147]. A recent analysis of genome‐scale CRISPR screening in a large group of cancer cell lines provided evidence that there may be hundreds of effective drug targets, but the vast majority is context‐specific [148]. Additionally, some essential genes have been associated with human diseases. For example, by population analysis‐based essential gene identification, genes intolerant to loss‐of‐function mutations are primarily essential in humans and have been found to play a role in human meiotic recombination, potentially contributing to the occurrence of certain diseases [149]. Furthermore, information on gene essentiality has been utilized to prioritize potential pathogenic variants of unknown disease‐related genes in human sequencing studies [150].

However, most of the existing essential genes have been identified in vitro, and significant differences between the genes essential for in vitro and those in vivo growth may be observed. An innovative solution is to perform screening in unconventional growth media such as acetate diversion, targeting *Pseudomonas aeruginosa* involved in lung infections. The screening workflow prioritizes compounds that exhibit activity in nutrient‐limited media [151]. Disease screening in host models is another approach to discovering in vivo targets. For example, high‐content microscopic screening of macrophages infected with *M. tuberculosis* identified a series of lead compounds targeting cytochrome *c* [152]. Although systematic investigations of genes have revealed numerous potential targets, a clear understanding of their functions is often inherent in the target validation process, which poses challenges in the identification of drug targets. Furthermore, the continuous evolution of drug resistance is inevitable, and further understanding of the resistance mechanisms is required. Finally, it must be acknowledged that gene essentiality is an evolvable trait, and genes associated with maximal importance may serve as promising drug targets. This poses difficulties in the search for drug targets but also brings opportunities. Therefore, more investigation is needed to fully define the importance of genes in different contexts and timescales and to further explore the genetic background of essential genes.

#### Introduction of the DEG database

**Figure 2:**
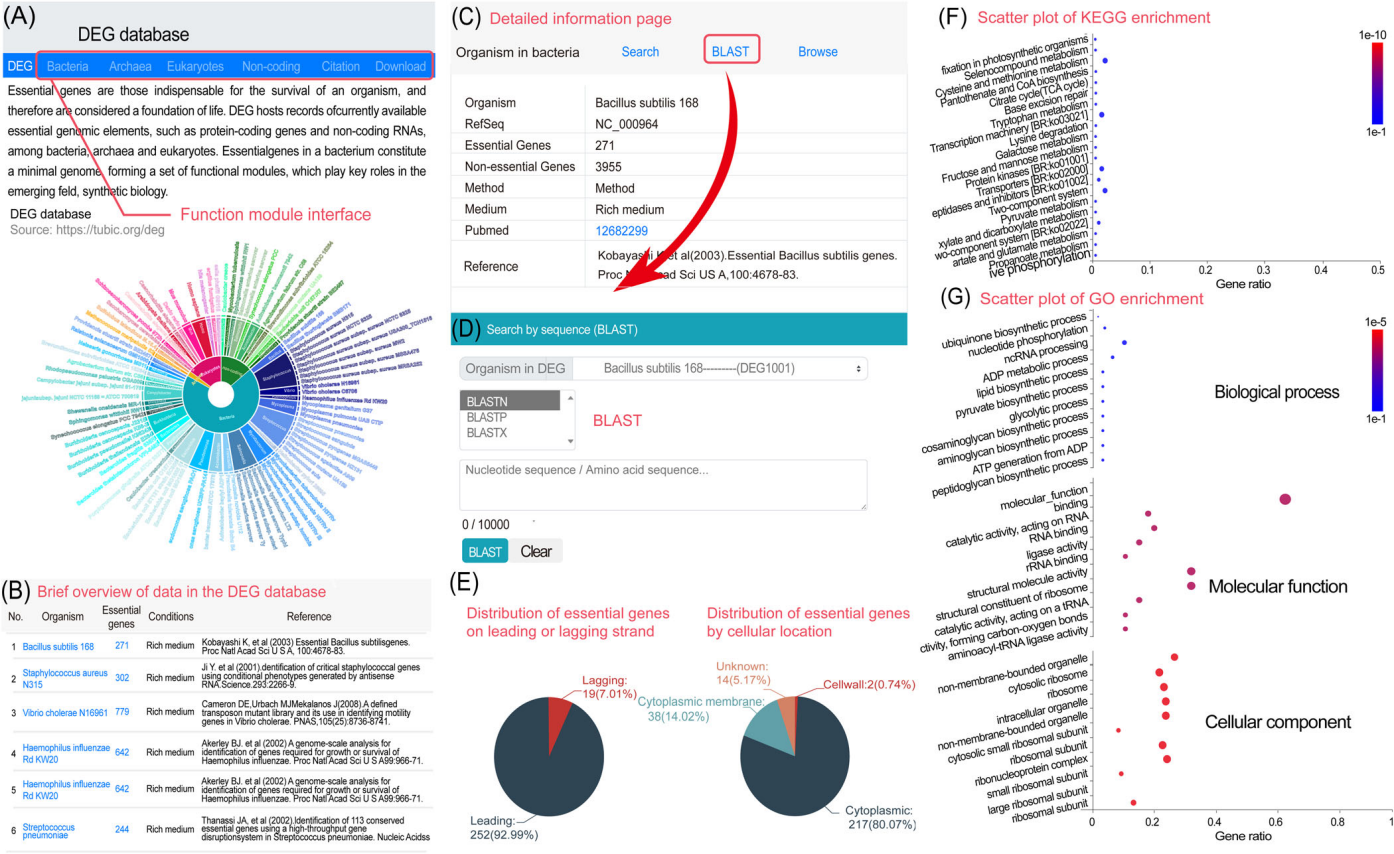
Screenshots and examples of user cases in the database of essential genes (DEG). (A) Homepage of the DEG database. Interfaces to other modules are supplied and the links to experimental results information for multiple essential genes in different species. (B) The summary page of all data within DEG. (C) A screenshot of the relevant information for an experimental result of a specific strain, including strain information, cultivation conditions, and reference citations. (D) The BLAST search interface for the corresponding strain. (E) The distribution of essential genes on the leading strand/lagging strand in the corresponding strain. (F) The Kyoto Encyclopedia of Genes and Genomes analysis results for essential genes in the corresponding strain. (G) The Gene Ontology analysis results for essential genes in the corresponding strain.

With the growing amount of essential gene data, there is an urgent need to organize them into a database to facilitate the use of these data. Thus, we established DEG in 2004 and updated it continuously with the accumulation of experimental data [15, 16, 17, 18]. This database compiles genome‐wide essential gene data obtained from a diverse range of experimental methods. The latest version of DEG 15.0, which encompasses bacteria, eukaryotes, and archaea, was released in 2021 (https://tubic.org/deg). Overall, there are 78, 35, and two essential gene sets for bacteria eukaryotes, and archaea determined by different experimental methods were stored in DEG 15.0 (Figure 2A,B), and displayed the specific information for each group of essential genes (Figure 2C). Moreover, DEG also comprises analysis modules associated with essential genes, particularly for those in bacteria, which contain the following functions: (i) The distribution of essential genes on the leading or lagging strands. (ii) The subcellular localization distribution of essential genes (Figure 2E). (iii) Orthologous groups, EC number [153], KEGG pathway [154], and GO [155] information on the essential genes (Figure 2F,G). (iv) A customized BLAST search tool allows users to conduct species‐ and experiment‐specific searches to identify essential genes in annotated or unannotated genomes (Figure 2D) [156]. In addition to essential genes, this database also collects experimental results for a subset of nonessential genes, as well as basic genetic elements other than protein‐coding genes, such as noncoding RNAs and replication origins [157, 158, 159, 160].

#### Applications of the DEG database

**Figure 3:**
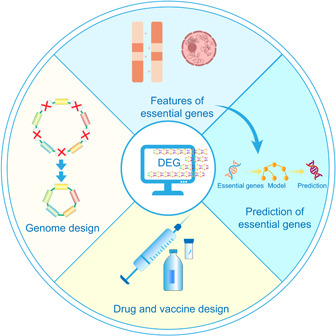
Main applications of the database of essential genes (DEG). The applications of DEG primarily focus on the following four areas: artificial genome design and construction, drug and vaccine design, essential gene feature analysis, and the prediction of essential genes.

DEG has become an important reference for research related to essential genes, and a “golden set” of the most popular databases featured in NAR database issues [161]. Information on essential genes for specific species can be quickly accessed using DEG [2]. Currently, the applications of DEG primarily focus on the following four areas: artificial genome design and construction, drug and vaccine design, essential gene feature analysis, and the prediction of essential genes (Figure 3).

Studies using essential data from DEG for genome design are typically top‐down approaches because nonessential regions of the genome are often considered candidates for deletion. In the top‐down approach, essential/nonessential gene data from the database are usually used to identify the location of the deletion in the genome to increase its stability [162, 163, 164]. Generally, regions containing nonessential genes are targeted for deletion, whereas regions containing essential genes are typically conserved. In addition, transporter genes, insertion sequences (ISs), toxin‐antitoxin pairs, and other functional elements have also been designed for deletion [165], depending on the research purpose. For example, Liu et al. selectively deleted genes from *Burkholderiales* strains to optimize their genome structure and growth rate [166]. This study used nonessential gene data from DEG to facilitate the identification of highly conserved genome‐reduced strains, which enhanced the predictability of genome engineering strategies and improved the efficiency and stability of strain production. In fact, the data from DEG can provide valuable references for bottom‐up genome construction. Therefore, DEG has been recommended as a reference resource for studying the minimal gene set required for bacterial survival [167, 168, 169].

Using a computational strategy to identify potential drug targets rather than culturing the entire microbe can significantly reduce time and cost. Gene information from DEG was used to identify essential proteins of pathogenic bacteria. To date, this method has been applied for the determination of potential drug targets in pathogens, including *Yersinia pseudotuberculosis* [170], *E. coli* [171], *P. aeruginosa* [172], *Shigella sonnei* [173], *Enterococcus faecium* [174], *Streptococcus pneumoniae* [175] and *Corynebacterium pseudotuberculosis* [176]. In addition, this process is applicable to vaccine design, known as reverse vaccinology [177]. Reverse vaccinology can significantly accelerate vaccine development as it can reduce the need for extensive empirical testing of individual antigens. Using this strategy, potential vaccine targets for *Helicobacter pylori* [178], *Brucellosis* [179], *Salmonella typhi* [180], and *Staphylococcus aureus* [181] have been identified. Furthermore, the vaccinology design pipelines, VacSol and PanRV, have been augmented by a process that employs DEG data to detect essential genes [182, 183].

DEG provides valuable data for the analysis of essential gene features, thus contributing to the field of essential gene characterization. The inclusion of nonessential gene data in the database enables comparative studies between the two categories. Numerous studies have already leveraged the essential gene features provided by DEG. For example, Luo et al. conducted an evolutionary conservation analysis of bacterial genomes and found that essential genes evolved more slowly than nonessential genes [104]. Essential genes with important functions such as translation, transcription, and replication are more critical and widespread in the leading strand than other functional subtypes of essential genes [101]. Surprisingly, if a specific functional domain of a protein is present in multiple organisms, its likelihood of essentiality increases [111]. Moreover, the metabolic networks of many microorganisms have been reconstructed based on essential genes and, to some extent, managed using automated systems such as the SEED [184] and BiGG [185] models. Based on data from DEG and other sources, Magnúsdóttir et al. systematically analyzed metabolic interactions within the gut microbiota as well as the influence of external factors on these interactions [186].

In the prediction of essential genes using machine learning methods, DEG provides an ideal training data set. For example, Guo et al. used a support vector machine to predict the integrity of human genes based on lambda‐interval *Z*‐curve features derived from nucleotide sequence data [187]. Zeng et al. constructed a deep learning‐based framework for essential gene prediction using PPI networks, gene expression data, and subcellular location information [120]. DeepHE predicted essential human genes by integrating features from both sequence data and PPI networks [188]. Shi et al. proposed a method called iEsGene‐CSMOTE for the machine learning method based on Support Vector Machines for identifying essential genes, which introduced a clustering‐based synthetic minority oversampling technique, that is, CSMOTE, to overcome the issue of imbalanced data [189]. In addition to utilizing essential gene features, Zhou et al. proposed an algorithm based on image recognition to predict essential genes, which utilized a convolutional spiking neural network with a learning rule called R‐STDP. The frequency codon group recognition images of essential and nonessential genes from DEG were used to predict essential genes [190].

### Analysis based on the DEG database

#### Comparison between essential and nonessential genes

**Figure 4:**
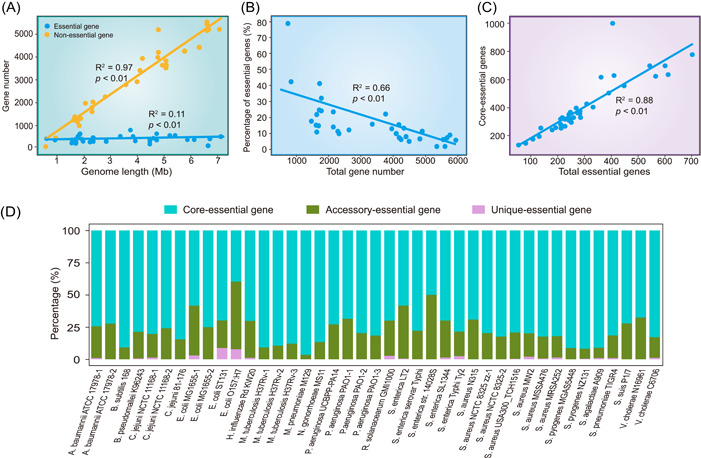
Relevant analysis results based on essential genes in the database of essential genes (DEG). (A) Numbers of essential and nonessential genes against genome length. (B) The percentage of essential genes against the total gene number. (C) The relationship between the number of core‐essential genes and total essential genes. (D) Distribution of essential genes under the pan‐genomic framework. The *x*‐axis represents different species. The essential genes existing in the unique, accessory, and core gene sets are highlighted in pink, green, and blue, respectively. The *y*‐axis represents the proportion of different types of essential genes. Those with multiple experimental results are labeled with a sequential number appended to the strain name, which was generated by ImageGP [192].

Because DEG 15.0 contains a significant quantity of essential gene and nonessential gene datasets of bacteria that have undergone genome‐wide gene essentiality screening, it presents an opportunity to explore the correlation between genome size and the number of essential genes. Certain essential genes, such as those involved in replication, transcription, and translation, encode fundamental cellular functions that are mandatory for all genomes irrespective of their size [191]. Therefore, we performed statistical analysis of the data in DEG that contained both essential and nonessential genes. Linear regression results showed a positive correlation between the number of nonessential genes and genome size, while the number of essential genes remained essentially constant (Figure 4A). Regardless of the genome size and experimental conditions, no bacterial species had more than 1000 essential genes. The percentage of essential genes, relative to the total number of genes, decreased as the total number of genes increased (Figure 4B). This result indicates that the number of essential genes remained relatively constant and did not change with genome length, whereas the number of nonessential genes was positively correlated with genome length.

Subsequently, we performed a statistical analysis of the COG annotation results between essential and nonessential genes in DEG. The proportions of each COG category for essential and nonessential genes are represented in blue and orange, respectively. Specifically, there were more essential than nonessential genes in categories C (energy production and conversion), F (nucleotide transport and metabolism), H (coenzyme transport and metabolism), J (translation, ribosomal structure, and biogenesis), K (transcription), and O (posttranslational modification, protein turnover, and chaperones). However, in categories G (carbohydrate transport and metabolism), N (cell motility), M (cell wall, Membrane, Envelope biogenesis), and U (intracellular trafficking, secretion, and vesicular transport), the number of nonessential genes was higher than that of the essential genes (Figure S1). In conclusion, the COG functional enrichment analysis suggests that essential genes tend to cluster in fundamental life processes, whereas nonessential genes play diverse roles and functions in environmental adaptation and substance synthesis.

### The distribution of essential genes in pan‐genomic classification

The concept of “pan‐genomics” was coined by Tettelin in 2008, which has allowed for the study of genome at the species level. “Pan‐genome” refers to a collection of all genes of a species, which can be categorized as “core genes” shared by all strains, “accessory genes” shared by two or more strains, and “unique genes” specific to certain strains [193]. Constructing a pan‐genome enables the identification of the diversity and composition of a bacterial species beyond the limitations of a single reference genome. The core genes in pan‐genomic classification are those shared by all individuals within a species and often perform important functions that encode basic biological and phenotypic relevance [194]. Considering the importance of essential and core genes in organisms, pan‐genome classification has been incorporated into present essential gene research. For example, by employing manual curation and a predictive model, Saxena et al. have successfully identified key essential gene sets of *Oleidesulfovibrio alaskensis* G20 using the sulfate‐reducing bacterium model and classified these genes through pan‐genome analysis. The results indicated that most essential genes belonged to the core gene category, whereas essential genes in other categories may be environment‐specific [195]. Furthermore, Wu et al. proposed a strategy to reduce the genome size of *B. subtilis* while simultaneously ensuring the retention of core and essential genes [196].

However, there is a lack of large‐scale pan‐genomic classification, specifically for essential genes across different species, to estimate the degree of overlap. Therefore, we propose a classification method for essential genes using a pan‐genomic framework. We downloaded the complete bacterial strains available on NCBI for the species included in DEG. To ensure the accuracy of pan‐genome analysis, 17 species with more than 70 complete genomes were selected for subsequent analysis. First, we filtered out sequences containing more than 1% N nucleotides. Next, we excluded strains with Average nucleotide identity (ANI) values <95% for the same organism. ANI refers to the average base similarity between homologous segments of two microbial genomes. ANI values between organisms belonging to the same species are typically ≥95% [197]. Ultimately, we obtained 5900 strains related to 17 bacterial species for pan‐genome analysis. We conducted a pan‐genome analysis and obtained results for the pan‐genomes (including core, accessory, and unique genes) of 17 species. By comparing the results with the essential gene data for the corresponding strains in DEG using BLAST, we determined the distribution of essential genes in the pan‐genomic classification (Figure 4D and Table S1).

The results showed that the majority of essential genes in bacteria belonged to core genes, except for *E. coli* O157:H7. This suggests that the essential genes and core genes that play important roles are largely overlapped; these types of genes are typically more conserved, which are hereinafter referred to as core‐essential genes. Linear regression analysis revealed a significant correlation between the number of core‐essential genes and the total number of essential genes (Figure 4C). However, some essential genes did not belong to the core genes, which may be related to the adaptation of the strains to specific growth conditions. Therefore, this classification would provide new insights into the function of essential genes. Moreover, the integration of pan‐genomic analysis with essential gene studies provides a new perspective for the development of vaccines and drugs. Because the core‐essential genes dictate the biological characteristics of pathogens to a greater extent, identifying core‐essential genes could be beneficial for devising efficacious broad‐spectrum drugs. By contrast, essential genes specific to certain pathogens may serve as potential targets for strain‐specific drugs.

## CONCLUSION AND FUTURE PERSPECTIVES

Technological advancements have facilitated large‐scale whole‐genome screening, providing valuable insights into the molecular basis of the essential genes involved in many biological processes. This reveals the complex and multifaceted nature of essential genes, opening up possibilities for their applications in synthetic biology and medicine. The generation of massive amounts of data has also led to the emergence of related online services such as databases and tools, which serve as references for future research. However, as discussed in this review, the essential gene is not an absolute concept, which depends on specific environments and contexts. Owing to the complexity of molecular interactions, comprehensively studying various aspects of essential genes using computational approaches, particularly machine learning methods, has proven to be promising. In the near future, the characterization of cells with diverse rational designs of genomes or genes will accelerate the generation of big data on the correlation between genomic content and cellular functionality. This will ultimately broaden our understanding of the entire cellular system and contribute to the application of essential genes in various fields. Furthermore, we are committed to continuously updating DEG. In addition to gathering experimental data on essential genes, we will extend our focus to the latest research findings and emerging trends pertaining to essential genes. We have a comprehensive plan to enhance and refine the database in the following areas. First, we aim to evaluate the degree of essentiality more effectively. In upcoming versions of the database, we will emphasize context dependence by annotating conditionally essential genes identified through experiments. Their significance was further stratified based on the likelihood of their existence under different experimental conditions. In addition, we will explore the provision of cross‐species links for corresponding essential genes, facilitating easy access to information on the shared presence of essential genes across diverse strains or species. Moreover, we will also add a pan‐genomic analysis module for essential genes, which will categorize essential genes into core, accessory, and essential categories in a pan‐genomic framework, to better study specific genes at the species level. Finally, we intended to augment the database with more additional features related to essentiality, including PPI data, expression profiles, and potential drug targets. Furthermore, suitable visualizations were employed to present the obtained results in an intuitive manner. Based on this series of improvements, we hope DEG could provide a more comprehensive and valuable reference for research on essential genes.

## AUTHOR CONTRIBUTIONS

Ya‐Ting Liang drafted the manuscript. Ya‐Ting Liang performed the major part of the data analyses. Feng Gao, Hao Luo, and Yan Lin conceived this study and reviewed, edited and refined the manuscript. Feng Gao supervised this project. All authors have read the final manuscript and approved it for publication.

## CONFLICT OF INTEREST STATEMENT

The authors declare no conflict of interest.

## Supporting information


**Figure S1**: The proportion of essential and non‐essential gene categories in COG classification.

**Table S1**: Distribution of essential genes under the pan‐genome classification.

## Data Availability

The DEG database is available at https://tubic.org/deg. Supporting Information materials (figures, tables, graphical abstract, slides, videos, Chinese translated version, and update materials) may be found in the online DOI or iMeta Science https://www.imeta.science/. The data that supports the findings of this study are available in the supplementary material of this article.
