# Supplementary material for: Recent advances in the characterization of essential genes and development of a database of essential genes
Source: Imeta. 2024 Jan 2;3(1):e157. doi: 10.1002/imt2.157 (PMC10989110; doi:10.1002/imt2.157)
Supplement: Supplementary file 1 — Figure S1: The proportion of essential and non‐essential gene categories in COG classification. [file IMT2-3-e157-s001.docx]

Supporting information to

Recent advances in characterization of essential genes and development of the database of essential genes

**Running title: Advances in essential gene characterization and database development**

Ya-Ting Liang^1^, Hao Luo^1^, Yan Lin^1^, and Feng Gao^1, 2, 3*^

^1^ Department of Physics, School of Science, Tianjin University, Tianjin, China

^2^ Frontiers Science Center for Synthetic Biology and Key Laboratory of Systems Bioengineering (Ministry of Education), Tianjin University, Tianjin, China

^3^ SynBio Research Platform, Collaborative Innovation Center of Chemical Science and Engineering (Tianjin), Tianjin, China

*Correspondence: [fgao@tju.edu.cn](mailto:fgao@tju.edu.cn) (Feng Gao)


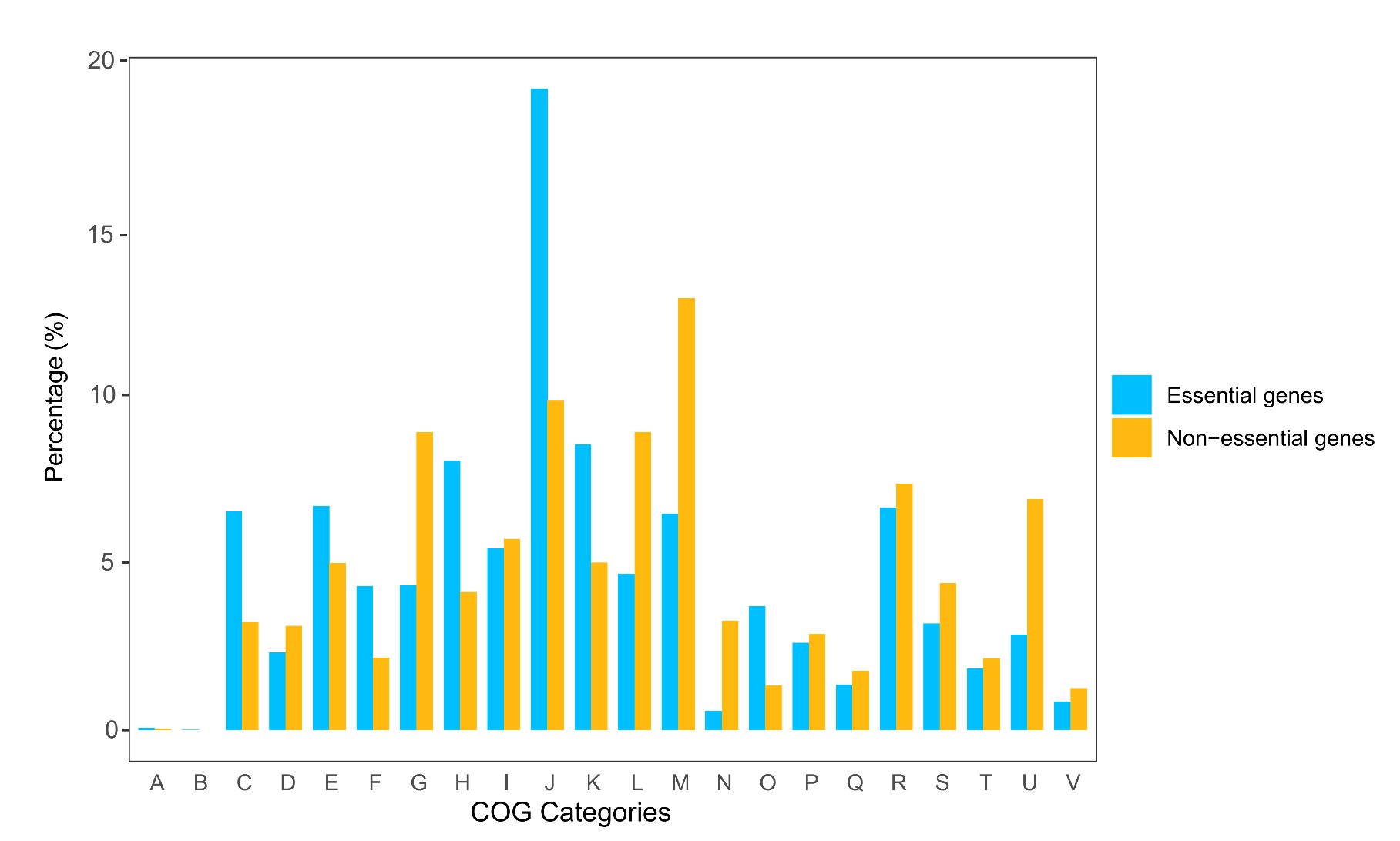


**Figure S1 The proportion of essential and non-essential gene categories in COG classification.** A-G represents different categories within the COG classification: A (RNA processing and modification), B (chromatin structure and dynamics), C (energy production and conversion), D (cell cycle control, cell division, chromosome partitioning), E (amino acid transport and metabolism), F (nucleotide transport, and metabolism), G (carbohydrate transport and metabolism), H (coenzyme transport, and metabolism), I (lipid transport and metabolism), J (translation, ribosomal structure, and biogenesis), K (transcription), L (replication, recombination, and repair), M (cell wall, membrane, envelope biogenesis), N (cell motility), O (posttranslational modification, protein turnover, and chaperones), P (inorganic ion transport and metabolism), Q (secondary metabolites biosynthesis, transport, and catabolism), R (general function prediction only), S (function unknown), T (signal transduction mechanisms), U (intracellular trafficking, secretion, and vesicular transport), V (defense mechanisms).
